# Automated parcellation and atlasing of the human subcortex with ultra-high resolution quantitative MRI

**DOI:** 10.1162/imag_a_00560

**Published:** 2025-04-29

**Authors:** Pierre-Louis Bazin, Josephine M. Groot, Steven Miletic, Lysanne Groenewegen, Anne C. Trutti, Martijn J. Mulder, Birte U. Forstmann, Anneke Alkemade

**Affiliations:** Full brain picture Analytics, Leiden, The Netherlands; Institute for Psychology, UiT - The Arctic University of Norway, Tromsø, Norway; Integrative Model-Based Neuroscience Research Unit, University of Amsterdam, Amsterdam, The Netherlands; Cognitive Psychology Unit, Institute of Psychology, Leiden University, Leiden, The Netherlands; Department of Experimental Psychology, Helmholtz Institute, University of Utrecht, Utrecht, The Netherlands

**Keywords:** subcortex, 7 tesla MRI, probabilistic atlasing, automated brain parcellation

## Abstract

Brain mapping efforts are time-consuming and benefit from an incremental approach. This allows periodic updates with newly acquired data, analysis methods, and resulting atlases and automated delineation approaches. Here, we present a new release of the Amsterdam Ultra-high field adult lifespan database (AHEAD). The AHEAD dataset is now extended with 105 7 Tesla slab quantitative MRI contrasts covering the subcortex at a 0.5 mm isotropic resolution. The data were collected from 105 participants covering the adult lifespan together with the previously released whole-brain acquisitions. Whole-brain and slab data now have been co-registered, manually delineated, and used for the expansion of the Multi-contrast anatomical subcortical structures parcellation (MASSP) algorithm, which now allows the individual delineation of 19 additional anatomical structures. MASSP2.0 can be used to delineate 35 structures in individual brain contrasts, creating a total of 63 created masks. Quantitative MRI maps, derived probability maps for anatomical structures, as well as MASSP2.0 are now freely available for further analyses.

## 1 Introduction

Brain atlasing was originally applied to uncover the microstructure of the human brain (Brodmann, 1909), which continues to be a primary goal for neuroanatomists. Over the years, a related major goal has become to develop atlasing tools that allow the interpretation of brain activation signals of various types of human behavior, which can be studied in a growing body of functional magnetic resonance imaging (fMRI) datasets (Forstmann et al., 2017). Data-sharing facilitates scientific research and creates the opportunity for research groups to work on their brain structure of interest, without the need to invest in the collection of high-quality research data (Candela et al., 2015;Poldrack & Gorgolewski, 2014).

Modern approaches to share brain data resources comprise the open availability of MRI datasets for reuse within the scientific community, and the use of reference spaces such as the Montreal Neurological Institute (MNI)-spaces (Fonov et al., 2009). These approaches allow the combination of datasets across imaging modalities, and the use of mixed methodology (e.g., a combination of histology and MRI) for building atlases (Alkemade et al., 2023). Scientists investigating brain structure-function relationships can use the anatomical information that is provided via atlases, or using delineation algorithms that allow automatic outlining of individual brain structures to help provide anatomical detail in their analyses (Holmes et al., 2015;Sudlow et al., 2015;Van Essen et al., 2012). To facilitate this research further, existing brain datasets can be extended incrementally, and used to create a growing body of anatomical information, and to derive atlases that can be updated with new structures in time. Creating such incremental atlases has several practical advantages, since the development of manual parcellations is a painstaking effort with an investment of hundreds of hours of individual researchers, and the completion of a whole-brain probabilistic atlas for MRI purposes will take many years (Alkemade et al., 2013). Additionally, the public sharing of databases is an efficient way to facilitate atlas improvement by creating data accessibility for research groups that have the expertise to develop algorithmic approaches, particularly if data are presented in a standardized manner through the use of the Brain Imaging Data Structure (BIDS) (Gorgolewski et al., 2016).

The use of automated parcellation procedures for atlasing purposes further facilitates the analyses of large imaging efforts, including the Human Connectome Project (Van Essen et al., 2012). These efforts have resulted in thousands of MRI acquisitions that are available for researchers across the world (Van Essen et al., 2012). We have previously argued that consistency, and the use of multiple brains to capture interindividual variation is crucial, and that high-quality manual, gold standard parcellations are needed to form a solid basis for successful training of automated algorithms and for the validation of the application of algorithms on novel datasets (Alkemade et al., 2020;Alkemade, Mulder, et al., 2022;Bazin et al., 2020).

What structures can be delineated on MRI images depends on the visible contrast, as well as the available spatial resolution. MR sequences have been designed specifically to allow the calculation of quantitative (q)MRI contrasts from a single scan acquisition, which results in multiple, intrinsically aligned MRI contrasts that provide rich contrast information that facilitates delineation efforts (Caan et al., 2019;Weiskopf et al., 2013). This approach can be approximated using co-registered contrasts obtained from different scan acquisitions in the same brain. The use of multiple contrasts, therefore, allows further zooming in to brain areas rich in small brain structures, such as those located in the mid-, inter-, and hindbrain. These efforts help to improve the interpretation of fMRI signals in these areas. It is feasible that fMRI signals are falsely attributed to a (larger) neighboring structure that is available in MRI atlases, but in reality originates from a smaller structure that has not been mapped out as a consequence of partial voluming (de Hollander et al., 2015). Creation of more detailed MRI atlases is, therefore, expected to increase the quality of fMRI analyses throughout the scientific community.

We have invested time and efforts to develop a high-quality 7T MRI dataset, manual delineation procedures for training and validation purposes, and the creation of a semi-automated algorithm, named Multi-contrast Anatomical Subcortical Structure Parcellation (MASSP) that allowed the delineation of 17 individual subcortical structures (Alkemade et al., 2020;Bazin et al., 2020). The development of manual delineations is extremely time-consuming, but still represents the gold standard. Our efforts are primarily directed toward the delineation of subcortical structures, since the subcortex has been relatively understudied for many years, although today many research groups world-wide provide excellent work uncovering deep brain structures (e.g.,Adler et al., 2018;Bianciardi et al., 2018;Börner et al., 2021;Chakravarty et al., 2006;Ewert et al., 2018;García-Gomar et al., 2019,^2022^;Iglesias et al., 2015,^2018^;Pauli et al., 2018;Sadikot et al., 2011;Saranathan et al., 2021;Singh et al., 2021;Su et al., 2019;Yushkevich et al., 2015). These combined efforts include the creation of parcellation algorithms as well as detailed descriptions of anatomical delineations. The MASSP algorithm is trained on manual delineation data and follows a Bayesian multi-object approach, combining shape priors, intensity distribution models, spatial relationships, and global constraints (Bazin et al., 2020). Additionally, it explicitly estimates interfaces between structures based on a joint model derived from signed distance functions (Bazin et al., 2020). To reliably add increasingly small structures to our algorithm, we adapted our research pipeline to incorporate additional, 0.5 mm isotropic resolution slab data from the same participants with the existing whole-brain acquisitions shared in AHEAD (Alkemade et al., 2020). We, therefore, expect that with an extension of the number of structures incorporated in MASSP, this will lead to improved parcellation results for structures included in the original MASSP release, in addition to reliable parcellation of newly added structures.

Crucial for the reliable imaging and atlasing of individual subcortical structures is high spatial resolution using (largely) isotropic voxels (Mulder et al., 2019). We now share a 0.5 mm isotropic dataset with partial brain coverage. This dataset represents an extension of our previous work in which we presented whole-brain 0.64 x 0.64 x 0.70 mm^3^images (Alkemade et al., 2020;Keuken et al., 2022). Furthermore, using these we have extended our MASSP parcellation algorithm which now allows delineation of 19 additional individual subcortical structures (Bazin et al., 2020).

## 2 Methods

### 2.1 Participants

The healthy participants included in the current studies are identical to those included in the first release of the Amsterdam ultra-High Field Adult lifespan Database (AHEAD) (Alkemade et al., 2020). All participants provided written informed consent. Participants were aged 18–80 years with self-reported health at the time of inclusion. Data were acquired in the same run as the data described previously (Alkemade et al., 2020). Demographics are summarized inTable 1.

**Table 1.** Demographics of the participants included in the Amsterdam ultra-high field adult lifespan database.

Age (years)	*Female*	*Male*	Total
*18-30*	27	15	42
*31-40*	6	6	12
*41-50*	6	7	13
*51-60*	7	5	12
*61-70*	7	6	13
*71-80*	7	6	13
*Total*	60	45	105

Demographics were previously published inAlkemade et al. (2020).

### 2.2 MRI scanning

MRI scans were acquired at the Spinoza Center for Neuroimaging in Amsterdam, the Netherlands, using a Philips Achieva 7T scanner with the previously described multi-echo magnetization-prepared rapid gradient echo (MP2RAGEME) sequence (Caan et al., 2019). Quantitative R1 and R2* maps, Quantitative Susceptibility Mapping (QSM), and Proton Density (PD) Maps were derived. In our original release, we shared T1-weighted (T1w), T1 map, R1 map, T2* map, R2* map, and QSM data at a reconstructed voxel size of 0.64 x 0.64 x 0.70 mm^3^. Previous contrasts were weighted images. We have recalculated quantitative contrasts, and now share R1 maps, R2*maps, rPD maps, and QSM at a 0.64 x 0.64 x 0.70 mm^3^resolution.

Additionally, we processed a dataset with partial brain coverage at a higher resolution (0.5 mm isotropic). We applied the MP2RAGEME, an extension of the MP2RAGE sequence byMarques et al. (2010). It consists of two rapid gradient echo (GRE_1,2_) images that are acquired in a coronal plane after a 180° degrees inversion pulse and excitation pulses with inversion times TI_1,2_= [670 ms, 3377.7ms]. A multi-echo readout was added to the second inversion at four echo times (TE_1_= 4.6ms, TE_2,1–4_= 4.6, 12.6, 20.6, 28.6 ms). Other scan parameters include flip angles FA_1,2_= [7°, 8°]; TR_GRE1,2_= [8.1 ms, 32.13ms]; bandwidth = 337.6 MHz; TR_MP2RAGEME_= 6778 ms; acceleration factor SENSE_LR_= 2, 2.5; FOV = 240 × 220 × 64 mm; voxelsize = 0.5 × 0.5 × 0.5 mm^3^; acquisition matrix was 292 × 290; turbo factor (TFE) = 150 resulting in 151 shots; and total acquisition time was 19:31min. Of these slabs, we now share R1 maps, R2*maps, rPD maps, and QSM at a 0.5 mm isotropic resolution. MRI parameters were chosen to accommodate visualization of the subcortex, and a relatively conservative acceleration factor was chosen to ensure good image quality and acceptable noise levels. We did not perform any prospective motion correction.

### 2.3 Quantitative map reconstruction

The MP2RAGEME data were first denoised with LCPCA (Bazin et al., 2019), using both magnitude and phase. First and second inversion, first echo images were combined to estimate R1 maps from a look-up table (Marques et al., 2010), and R2* maps were computed by least-squares fitting of the exponential decay over the multi-echo images of the second inversion. Non-brain regions were removed by skull stripping of the second inversion, first echo image (Bazin et al., 2014). Relative proton density (rPD) maps were obtained from the MP2RAGEME model (Caan et al., 2019) and previously estimated R1 and R2* parameters. All processing was performed in Nighres (Huntenburg et al., 2018). For QSM, phase maps from the second, third, and fourth echoes of the second inversion were processed with TGV-QSM (Langkammer et al., 2015) and averaged. rPD maps were further processed with N4 in ANTs (Tustison et al., 2010) for removing B0 inhomogeneities. Quantitative R1, R2*, and QSM are intrinsically corrected for B0 inhomogeneities. B1 inhomogeneities are still present, but have a limited effect. Tests on B1 correction on a subset of subjects proved inconclusive, as noise in acquired B1 maps can also impact the results and corrections were not pursued further*.*The resulting high-quality images, and the coregistration of the data with the previously published whole-brain acquisitions are illustrated inFigure 1. Images were visually inspected for artifacts that could potentially interfere with delineations of subcortical structures. We would like to point out that some drop-outs occurred in the occipital regions, which did not affect subcortical atlasing.

**Fig. 1.**
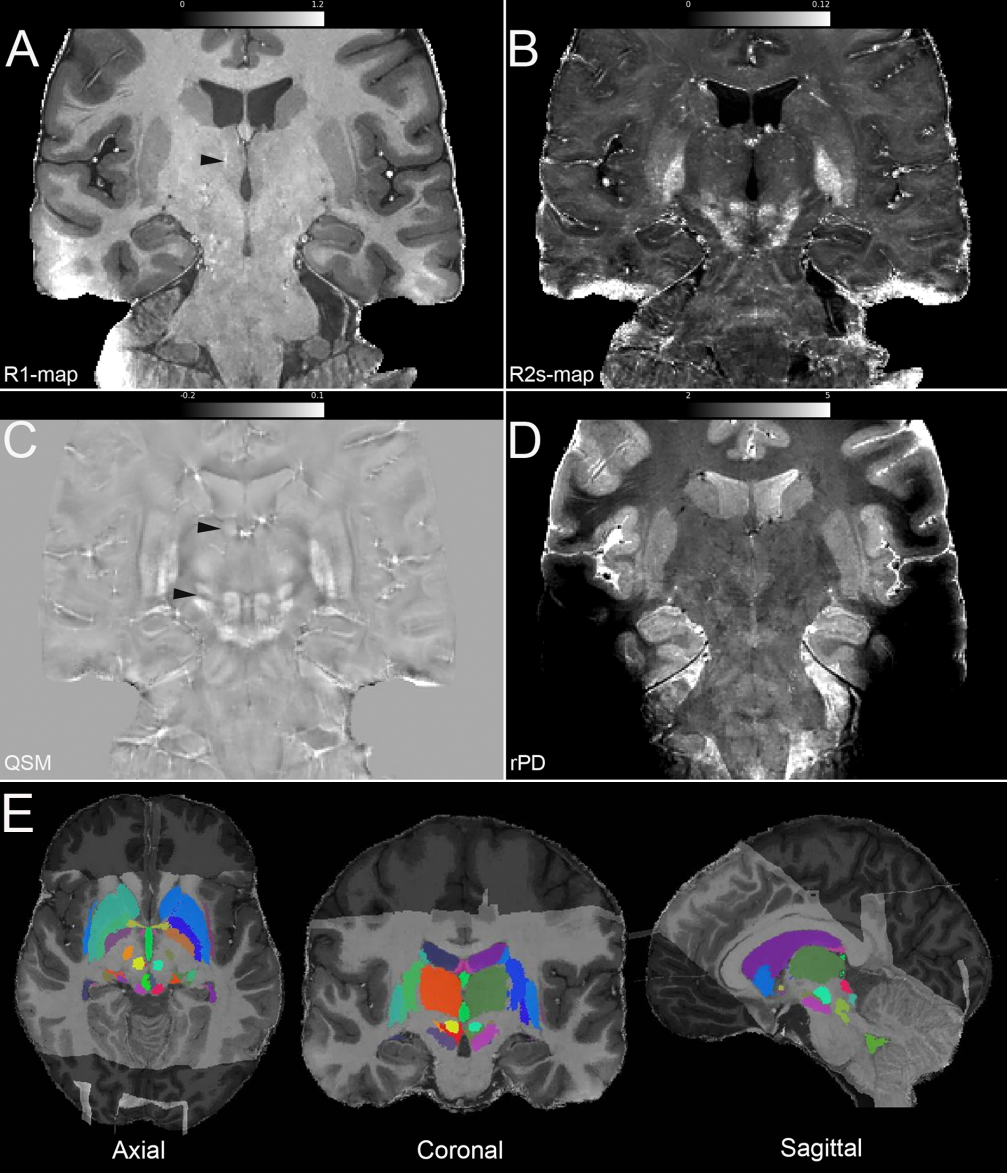
Example of the 0.5 mm isotropic 7T MRI data with partial brain coverage. The arrowhead in the R1-map (A) indicates the visibility of the thalamic lamina. (B) shows the R2*-map. In the QSM (C), you can distinguish the anteroprincipal nucleus of the thalamus as well as the border between the substantia nigra and the subthalamic nucleus can be distinguished (arrowheads). (D) shows the relative Proton Density (E) shows the combination of the slab with the whole-brain data and the delineations created with MASSP2.0.

The 0.64 x 0.64 x 0.70 mm^3^and 0.5 x 0.5 x 0.5 mm^3^datasets were used in combination to extend the number of structures included in MASSP through the addition of 16 new structures, and the replacement of the striatum with separate masks for the Caudate Nucleus (CAU), the Putamen (PUT), and the Nucleus Accumbens (NACC; seeTable 2).

**Table 2.** Brain structures included in MASSP2.0.

Structures in MASSP2.0	Abbreviation	Hemispheres/side
Subthalamic nucleus	STN	L/R
Red nucleus	RN	L/R
Substantia nigra	SN	L/R
Globus pallidus, pars interna	GPi	L/R
Globus pallidus, pars externa	GPe	L/R
Thalamus	THA	L/R
Ventricles		
Lateral	LV	L/R
3 ^rd^	3V	3
4 ^th^	4V	4
Amygdala	AMG	L/R
Internal capsule	ic	L/R
Ventral tegmental area	VTA	L/R
Fornix	fx	LR
Periaqueductal grey	PAG	L/R
Pedunculopontine nucleus	PPN	L/R
Claustrum	CL	L/R
**Inferior colliculus**	**ICO**	**L/R**
**Superior colliculus**	**SCO**	**L/R**
**Habenular complex/lateral habenula**	**LHb**	**L/R**
**Anterior commissure**	**ac**	**LR**
**Posterior commissure**	**pc**	**LR**
**Cholinergic forebrain nuclei**	**CHN**	**L/R**
**Dorsal Raphé nucleus**	**DRN**	**LR**
**Medial Raphé nucleus**	**MRN**	**LR**
**Caudate nucleus**	**CAU**	**L/R**
**Putamen**	**PUT**	**L/R**
**Nucleus accumbens**	**NACC**	**L/R**
**Cornu ammonis 1**	**CA1**	**L/R**
**Cornu ammonis 2/3**	**CA23**	**L/R**
**Dentate gyrus**	**DG**	**L/R**
**Pre/pro subiculum**	**PRESUB**	**L/R**
**Subiculum**	**SUB**	**L/R**
**Uncus**	**UNC**	**L/R**
**Lateral geniculate nucleus**	**LGN**	**L/R**
**Medial geniculate nucleus**	**MGN**	**L/R**

Structures included in MASSP2.0. Structures indicated in bold have been added to MASSP2.0.

Note that the striatum was present in the original MASSP release, and has been replaced by the Caudate Nucleus, Putamen, and Nucleus Accumbens. L = left, R = right, LR indicates masks crossing the midline.

### 2.4 Manual delineations

New structures were outlined in individual space by two independent raters in the ten individuals who were used for training the original MASSP algorithm (Bazin et al., 2020). Either whole-brain data or slab data were used (seeTable 3). Delineations were performed on MRI contrasts of the ten participants who were included in the previously defined training dataset for the MASSP algorithm (Bazin et al., 2020). An additional ten scans were manually delineated by two raters for validation purposes, to ensure that MASSP performs well in all age groups. A separate approach was taken for the striatal structures. Manual masks created previously (Alkemade, Mulder, et al., 2022) for the striatum were adapted to create masks for the NACC, CAU, and PUT.

**Table 3.** Overview (dilated) Dice scores of manual and automated delineations.

Structure	Hemi	WB or SB	Manual Dice	Manual dilated Dice	MASSP2.0 Dice	MASSP2.0 dilated Dice	MASSP2.0 boundary (mm)
Inferior colliculus	L	WB	0.83 (0.02)	0.98 (0.01)	0.67 (0.05)	0.90 (0.04)	0.54 (0.08)
	R	WB	0.84 (0.01)	0.99 (0.01)	0.68 (0.04)	0.92 (0.04)	0.53 (0.09)
Superior colliculus	L	WB	0.72 (0.03)	0.92 (0.02)	0.70 (0.11)	0.89 (0.11)	0.61 (0.26)
	R	WB	0.77 (0.03)	0.95 (0.02)	0.68 (0.10)	0.88 (0.09)	0.64 (0.22)
Habenular complex/lateral habenula	L	WB	0.81 (0.02)	0.98 (0.01)	0.37 (0.14)	0.69 (0.16)	1.04 (0.55)
	R	WB	0.80 (0.02)	0.98 (0.00)	0.40 (0.14)	0.72 (0.15)	0.96 (0.53)
Anterior commissure	LR	WB	0.75 (0.01)	0.96 (0.01)	0.64 (0.06)	0.91 (0.03)	0.44 (0.08)
Posterior commissure	LR	WB	0.58 (0.05)	0.87 (0.04)	0.50 (0.10)	0.85 (0.10)	0.49 (0.15)
Cholinergic forebrain nuclei	L	SB	0.71 (0.03)	0.91 (0.02)	0.68 (0.11)	0.90 (0.08)	0.52 (0.20)
	R	SB	0.72 (0.04)	0.91 (0.03)	0.71 (0.05)	0.92 (0.04)	0.92 (0.21)
Dorsal Raphé nucleus	LR	SB	0.59 (0.02)	0.90 (0.03)	0.53 (0.11)	0.81 (0.12)	0.71 (0.29)
Median Raphé nucleus	LR	SB	0.47 (0.04)	0.80 (0.05)	0.46 (0.11)	0.78 (0.10)	0.75 (0.34)
Cornu ammonis 1	L	WB	0.57 (0.03)	0.84 (0.03)	0.58 (0.11)	0.80 (0.11)	0.86 (0.29)
	R	WB	0.58 (0.03)	0.86 (0.03)	0.59 (0.08)	0.81 (0.07)	0.87 (0.19)
Cornu ammonis 2/3	L	WB	0.35 (0.03)	0.76 (0.05)	0.36 (0.08)	0.64 (0.13)	1.37 (0.93)
	R	WB	0.38 (0.02)	0.77 (0.03)	0.36 (0.09)	0.66 (0.08)	0.29 (0.42)
Dentate gyrus	L	WB	0.62 (0.03)	0.89 (0.02)	0.55 (0.17)	0.77 (0.17)	1.17 (1.13)
	R	WB	0.62 (0.03)	0.89 (0.02)	0.58 (0.09)	0.81 (0.06)	0.92 (0.26)
Presubiculum	L	WB	0.43 (0.05)	0.81 (0.04)	0.47 (0.07)	0.79 (0.06)	0.69 (0.12)
	R	WB	0.45 (0.03)	0.79 (0.03)	0.36 (0.11)	0.65 (0.12)	1.02 (0.27)
Subiculum	L	WB	0.63 (0.03)	0.86 (0.02)	0.68 (0.06)	0.87 (0.05)	0.74 (0.25)
	R	WB	0.61 (0.02)	0.86 (0.03)	0.60 (0.08)	0.81 (0.06)	0.88 (0.22)
Uncus	L	WB	0.67 (0.04)	0.90 (0.03)	0.63 (0.10)	0.82 (0.09)	1.17 (0.54)
	R	WB	0.68 (0.03)	0.91 (0.02)	0.62 (0.06)	0.81 (0.06)	1.17 (0.38)
Lateral geniculate nucleus	L	SB	0.75 (0.02)	0.94 (0.01)	0.65 (0.10)	0.88 (0.08)	0.60 (0.16)
	R	SB	0.73 (0.02)	0.91 (0.03)	0.57 (0.09)	0.81 (0.10)	0.76 (0.25)
Medial geniculate nucleus	L	SB	0.71 (0.02)	0.93 (0.01)	0.39 (0.18)	0.68 (0.23)	0.96 (0.55)
	R	SB	0.68 (0.07)	0.89 (0.06)	0.30 (0.17)	0.59 (0.21)	1.13 (0.45)

L/R = separate masks were created for each hemisphere. SB = slab, WB = whole-brain, LR = a single mask was created for midline (crossing) structures. (Dilated) Dice factors and boundaries are presented as mean (s.e.m.).

All manual delineations were performed in individual space. The delineation order of the scans and, when appropriate, the start hemisphere for delineations were randomized. Separate delineations were made for the left and right hemisphere if appropriate. The inclusion of separate masks for the left and right hemisphere in a majority of the structures led to a total number of 63 masks. Delineation protocols developed for this work have been described previously (Alkemade, Mulder, et al., 2022) or are available inSupplement 1, except for the delineations of the geniculate bodies which were based on the descriptions ofBianciardi et al. (2018), and the hippocampal subfields which were based on the description ofDalton et al. (2017).

### 2.5 Interrater agreement

To assess the quality of the delineations, we calculated Dice and dilated Dice measures in the ten participants included in the training set. Additionally, we performed manual delineations in an additional ten participants from various age groups to confirm appropriate performance of MASSP2.0 in all ages. The Dice coefficient (Dice, 1945) is based on the precise overlap between voxels in both delineations, whereas the dilated Dice coefficient allows the dilation of the mask of one voxel at the borders, which measures the overlap between delineations allowing for one voxel of uncertainty (Trutti et al., 2021). This is particularly relevant for, for example, elongated structures, which have a relatively high number of border voxels, and for small structures. Since we report on brain structures that vary substantially in size, the percentage of voxels located at the border of an individual brain structure varies substantially between the delineated structures. Since Dice factors were already determined for striatum as a whole, and the current masks for NACC, CAU, and PUT are based on those published previously, we did not include a second rater to determine Dice factors between manual raters for these structures.

### 2.6 Automated parcellation retraining

With the new structure delineations, we retrained the MASSP algorithm to include 19 new structures in addition to the original structures, resulting in a total of 35 individual structures separated into left and right hemisphere when relevant. The striatum was replaced by masks of the ACC, CAU, and PUT. A number of the new structures were delineated on high-resolution slab data, which were co-registered with the whole-brain images (seeTable 3). To do so, we co-registered individual slabs to the corresponding whole-brain data, and brought the combined images to the AHEAD template in MNI2009b space at 0.5 mm isotropic resolution. The slabs and whole-brain images were co-registered rigidly using the quantitative R1 images. Registration to MNI space was performed by co-registering all contrasts (quantitative R1, R2*, and QSM) for each individual whole-brain data set to the AHEAD template built from co-registration and averaging of whole-brain quantitative R1 maps to the MNI2009b T1-weighted template. Transformations were combined in order to maintain the 0.5 mm isotropic resolution of the slabs. When training, all contrasts were integrated into the model, as the slabs did not cover all structures fully. For instance, in some cases the rostral tip of the striatum was not included in the slab. In a given voxel, a subset of the contrasts may be undefined due to artefacts, masking, or the location of the voxel outside of the imaging slab. MASSP2.0 automatically builds the likelihood from the product of the available contrasts available of each voxel, weighted by the corresponding inverse exponent placing all posterior on the same scale. The intensity modeling in MASSP was thus adjusted to ignore undefined contrasts, so only the available information was used in the model. The resulting MASSP parcellation priors include intensity models for R1, R2*, QSM and relative proton density (rPD) at 0.64 x 0.64 x 0.70 mm^3^and 0.5 mm isotropic resolution. Like the original MASSP algorithm, the version trained with the new priors followed a Bayesian estimation procedure, building separate posterior probabilities for each structure and their interfaces first combining spatial location priors, shape skeleton priors, and matching of image intensities with contrast priors; then applying probability diffusion, topology correction, and volume prior constrained region growing to generate a maximum a posteriori parcellation and voxel-wise posterior probability map (seeBazin et al., 2020for algorithmic details)*.*Similarly to the manual delineations, Dice and dilated Dice overlap of the automated and primary manual delineations were computed for each structure. To render training and testing independent, we used a leave-one-out approach building MASSP priors on 9 subjects and applied it to the left out subject. This process was performed 10 times.

### 2.7 Updating the MASSP subcortical atlas

Finally, the new delineations and automated MASSP parcellation method allowed the reprocessing of the AHEAD database to build a new version of the MASSP subcortical atlas including 35 structures. As automated parcellations were performed directly in MNI2009b space, probabilistic atlas maps were built by direct averaging of the structure binary masks across the 105 database subjects (lifespan atlas), or in age-based subgroups (young: 18–40 years, middle-aged: 40–60 years, older: 60–80 years). An average parcellation was obtained through splitting the maximum probability parcellation into individual binary masks for each structure, and averaging over the group.

## 3 Results

We have extended MASSP allowing the automated delineation of 35 individual structures listed inTable 2. Overall, the automated delineations approximated the corresponding manual delineation, comparable to the performance of the original MASSP algorithm. The dilated Dice overlap was generally above 0.75, with the exception of the lateral habenula (LH), Cornu Ammonis (CA)2/3, presubiculum (presub), and medial geniculate nucleus (MGN). The majority of average boundary distances fall below 1 mm. Delineation performance for previously reported structures was slightly higher in terms of overlap, and lower for boundary distances, as a result of the use of higher-resolution data compared to the original MASSP (seeSupplement 2).

The new subcortical atlas based on the processing of the entire extended AHEAD database is presented inFigure 2and available on FigShare (seeData and Code Availabilitysection).

**Fig. 2.**
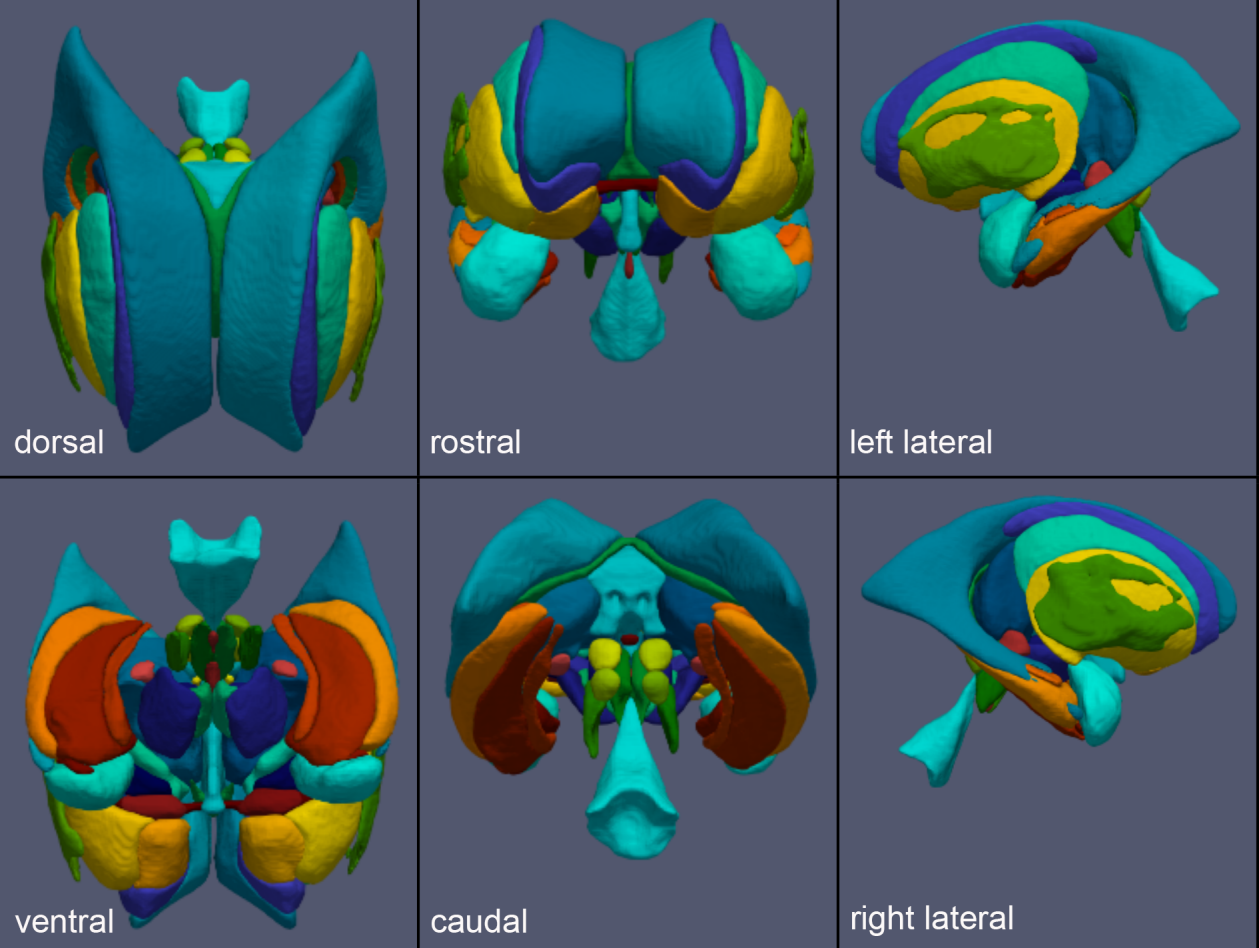
3D representations of the MASSP2.0 subcortex atlas.

The algorithm is integrated into the Nighres toolbox (Huntenburg et al., 2018), and can use the original or updated set of structures. With this release, we have included additional contrast priors for the Multi Parameter Mapping (MPM) sequence at 3T and 7T. The MASSP2.0 updated atlas is shared openly via Figshare. We also share the qMRI data of whole brain and slab acquisition of the AHEAD database, providing R1, R2*, rPD maps, and QSM. Data are organized in a BIDS-like format, and available via Figshare (seeData and Code Availabilitysection).

## 4 Discussion and/or Conclusions

As the demand for high-resolution brain atlases continues to grow, tools and resources that allow precise mapping of anatomical structures are crucial for advancing our understanding of the human brain across the lifespan. Here, we provide the third release of the AHEAD dataset, which now contains 7 Tesla structural qMRI data, including a whole-brain coverage 0.64 x 0.64 x 0.70 mm^3^dataset, a 0.5 x 0.5 x 0.5 mm^3^slab, showing the diencephalon and the brain stem, as well as parts of the neocortex, and a series of diffusion MRI scans obtained at 3 Tesla (Alkemade et al., 2020;Keuken et al., 2022). The 7 Tesla slab data were co-registered with whole-brain data from the same participants. 7 Tesla data were acquired in a single scan session. We would like to point out that we designed the slab acquisition to allow the delineation of subcortical structures, and that we observed some signal-dropout in the occipital regions (Fig. 1), which did not interfere with the creation of our delineations. Additionally, in some cases, the field of view did not cover the entire subcortex and the rostral extent of the striatum would not be included. This did not have any repercussions for the delineations since the striatum was delineated on the whole-brain data. The slab scans were used for the delineation of increasingly small deep brain structures, including MRN and DRN.

The whole-brain acquisition obtained from the same scan session and published previously allows the coregistration with whole-brain data. These efforts represent an extension of our previous work atlasing the human subcortex (Alkemade et al., 2020;Alkemade, Mulder, et al., 2022;Bazin et al., 2020). The combination of the MRI contrasts creates possibilities for the mapping of smaller deep brain structures, such as a subset of the iron-rich nuclei of the Raphé complex. Manual delineations were used to retrain and extend the MASSP algorithm, which we now share as MASSP2.0. The original MASSP and MASSP2.0 perform the same general steps: Co-registration of the individual data to the AHEAD template, and running the MASSP algorithm using the MASSP or MASSP2.0 atlas (see alsoSubcortex Parcellation — Nighres 1.4.0 Documentation, n.d.). MASSP2.0 includes an explicit model of quantitative R1, R2*, QSM, and PD derived from the MP2RAGEME sequence. It will generally perform well with quantitative maps derived from other sequences such as MPM at the same field strength, although differences on the quantitative modeling and artifacts may impact the result. Additionally, the learned contrasts can also be translated to other sequences by building a transfer function from co-aligned images of the MP2RAGEME and desired contrast (seeBazin et al., 2020for details). With this release, we have included additional contrast priors for the MPM sequence at 3T and 7T.

The inclusion of increasingly smaller structures, and the calculated interrater agreement measures (Table 3) call for a reevaluation of the applicability of Dice factor cut-off scores as indicators of delineation quality. We previously indicated that a Dice coefficient above 0.75 was considered a useful cut-off value for assessing acceptable quality (Alkemade, Mulder, et al., 2022). However, smaller structures have a relatively high number of voxels located at the border of anatomical structures, which are sensitive to partial voluming effects, which is reflected in lower Dice. Dilated Dice is, therefore, a better measure of interrater agreement in smaller and elongated structures (Trutti et al., 2021).

Extracting volume estimates from these increasingly small regions becomes a challenge, due to the limitations of MRI resolution and partial voluming effects. For instance, our reported label volume for the Dorsal Raphé Nucleus (DRN) was 63 mm^3^as determined using MASSP2.0 in individuals aged 19–30 years, a number which we compared to data available from literature. We found that the volume we report did not change with age, and is substantially smaller than the volumes reported in young individuals by Bianciardi et al. (345 mm^3^) (Bianciardi et al., 2015), and bySchain et al. (2013)who applied high-resolution research tomography and PET-scanning, and reported a DRN volume of 149 and 163 mm^3^, respectively. These differences could not be attributed to age differences, since ages were comparable across studies. Additionally, our current dataset does not show any indications for age-related volume alterations for the DRN across the adult lifespan (average DRN volume for the entire cohort was 62 mm^3^). These differences illustrate the need for an anatomical ground truth. We have argued previously that the combination of*post mortem*MRI with histology can provide this (Alkemade, Bazin, et al., 2022). Indeed, an older study has estimated the volume of the DRN in post mortem tissue at 71 mm^3^(Baker et al., 1990). The reported volumes of the MRN, including ours, also show substantial variation, and vary between 14 and 219 mm^3^(Bianciardi et al., 2015;Schain et al., 2013). Unfortunately, Baker et al. did not report MRN volumes (Baker et al., 1990). It is unclear to what extent the volume differences represent interindividual variation, or whether they result from methodological differences between these studies. The use of different contrast mechanisms (e.g., mapping of connectivity patterns vs. grey or white matter contrast) could lead to different atlasing results of the same structure. This would be the case if connections are limited to specific areas of an individual structure, or if the connections do not respect the same anatomical boundaries as the gray white matter contrast. The studies described above, providing estimates of the Raphé complex, depend on different contrast mechanisms. These differences could, at least in part, explain the large volumes reported for the DRN using PET tracing of serotonin transporters (Schain et al., 2013). Serotonergic neurons have been reported not to respect the histological boundaries of the DRN (Boldrini et al., 2005). The signal derived from serotonin tracers is, therefore, expected to extend beyond the classical boundaries of the DRN as well. Also, the volume difference may have been further exaggerated by the difference in spatial resolutions and signal specificity that can be obtained using the different techniques. The Raphé complex serves as an example here, but these challenges are not restricted to these specific nuclei. The question to what extent the applied methodology co-determines the volume of individual structures is relevant for all atlased structures. Additionally, the chosen parameters within an imaging modality can strongly influence volumetric measures. Modeling studies indicate that in the field of MRI, volume estimates can be influenced substantially by the available voxel size and shape (Mulder et al., 2019).

Our efforts contribute to the closing of the gap between the number of structures residing in the subcortex and the number of structures that are incorporated in atlases available for MRI applications (Alkemade et al., 2013). The advantage of using incremental approaches for atlasing efforts in the same dataset is that the applied algorithms can improve their performance by incorporation of new gold standard manual delineations. MASSP makes use of shape priors not only of the structures of interest, but also of distance information on structures located in close proximity. Therefore, the extension of these datasets with new structures is expected to improve the performance of the MASSP algorithm on previously included structures (Supplement 2). Many other valuable available atlasing initiatives invest in the creation of atlases for the analyses of increasingly small structures, including the individual nuclei of the thalamus (Su et al., 2019), and both cortical and subcortical atlases that are available in Freeserver (Fischl et al., 2002) and FSL-First (Patenaude et al., 2011). These atlases include different target structures, as well as different atlasing approaches, including gray and white matter contrast approaches, and connectivity-based atlasing.

We developed MASSP to allow individual delineations rather than atlas-based delineations, although we appreciate the challenges of contrast availability, and possible limitations in the generalization of the applicability of automated algorithms to any type of MRI contrast. We previously found that MASSP and other available approaches allow reliable delineation of structures on the HCP data. It is important to note that other datasets may have characteristics that do not allow the use of MASSP (Bazin et al., 2020). For contrasts on which MASSP does not perform delineations accurately, researchers can revert to the use of atlases, although we would like to call for caution. MASSP2.0 targets increasingly small structures. The correspondence between the location of the structure and its atlas location may be difficult to verify. Additionally, it is important to take into account that anatomical scans used for co-registration purposes in studies often have a different resolution as compared to the fMRI data acquisition. fMRI data are usually acquired at a lower voxel resolution as compared to structural MRI, which increases the chances of partial voluming effects being captured by the fMRI data. This may subsequently lead to the attribution of an activation pattern to the wrong anatomical structure (de Hollander et al., 2015). We expect that these challenges will at least partially be resolved in the future, with further improvement of image acquisition techniques.

In conclusion, we have shown previously that the MASSP algorithm performs well against other automated protocols, and the application to other MRI contrasts provides stable parcellations(Bazin et al., 2020). The new MASSP2.0 algorithm is available for reuse by other researchers and the 0.5 mm isotropic slab data have now been added to the AHEAD database, which has also been published previously (Alkemade et al., 2020).

Atlasing of small deep brain structures remains challenging. For larger subcortical structures, such as the individual nuclei of the basal ganglia or the thalamus as a whole, there is sufficient agreement on the anatomical borders to allow consistency across research groups. With the availability of increased anatomical detail, smaller structures, such as a subset of the individual nuclei of the (meta)thalamus, become visible. Interestingly, the availability of multiple interpretations of the individual thalamic nuclei illustrates the challenges in the field of brain atlasing (Mai & Majtanik, 2018). The strong differences in the interpretation of the thalamic anatomy emphasize the value of clear anatomical descriptions that underlie the creation of MRI atlases (Alkemade, et al., 2022;Dalton et al., 2017;García-Gomar et al., 2019;Zaborszky et al., 2008).

## Ethics

These studies were approved by the local Ethical Review Board of the Faculty of Social and Behavioural Sciences at the University of Amsterdam (2016-DP-6897).

## Supplementary Material

Supplement 1

Supplement 2

## Data Availability

The data described here are available via Figshare (Table 4). Per participant, we share slab contrasts covering the subcortex at a 0.5 mm isotropic resolution, in addition to the already available whole-brain data. The data are shared in the Nifti format which was used for manual delineations. Demographics of the participants are shared in a participants.csv file. Probabilistic atlases for the whole cohort and age groups are provided for each structure along with a summary parcellation in Nifti format. The MASSP2.0 algorithm is included in the open-source toolbox Nighres, along with an example processing script on a sample subject from the database. Figshare data.
