# Supplementary material for: Automated parcellation and atlasing of the human subcortex with ultra-high resolution quantitative MRI
Source: Imaging Neurosci (Camb). 2025 Apr 29;3:imag_a_00560. doi: 10.1162/imag_a_00560 (PMC12319765; doi:10.1162/imag_a_00560)
Supplement: Supplement 1 [file imag_a_00560-supp1.pdf]

Supplement 1: Manual delineation protocols

Automated parcellation and atlasing of the human subcortex with ultra high-resolution quantitative MRI

Pierre-Louis Bazin[1], Josephine M Groot[2], Steven Miletic[3,4], Lysanne Groenewegen[3], Anne C Trutti[3], Martijn J Mulder[5], Birte U Forstmann[3], Anneke Alkemade[3]

[1]Full brain picture Analytics, Leiden, The Netherlands
[2]Integrative Model-Based Neuroscience Research Unit, University of Amsterdam, Amsterdam, The Netherlands
[3]Cognitive Psychology Unit, Institute of Psychology, Leiden University, The Netherlands
[4] University of Utrecht, Psychology and Social Sciences, Utrecht, The Netherlands
Corresponding author: Anneke Alkemade, Integrative Model-based Cognitive Neuroscience Research Unit, University of Amsterdam, , Nieuwe Achtergracht 129B | Room G0.01, PO box 15926,1001 NK Amsterdam, The Netherlands

j.m.alkemade@uva.nl

Keywords:

Subcortex, 7 Tesla MRI, probabilistic atlasing, automated brain parcellation

This supplementary material contains the manual delineation protocols for the structures included in MASSP2.0. For structures also included in the original MASSP we refer to Alkemade et al, 2022 (Alkemade et al., 2022).

Inferior and superior colliculus (ICO) and SCO)

The ICO is an ellipsoid grey matter region located in the caudal half of the mesencephalic tectum, and the SCO is a gray matter nucleus located in its rostral half. Both structures are laterally bordered by the ventricular system (García-Gomar et al., 2019). Delineations are created on the R2* or QSM contrast on which they present as hyperintense structures. The red nucleus (RN) is used as an anatomical landmark for orientation. In the sagittal plane at the level of the RN, the SCO and ICO can be readily be discerned. The delineations are started at their central level in the axial plane. First the structures are delineated in the superior direction, followed by the delineation of the inferior part.

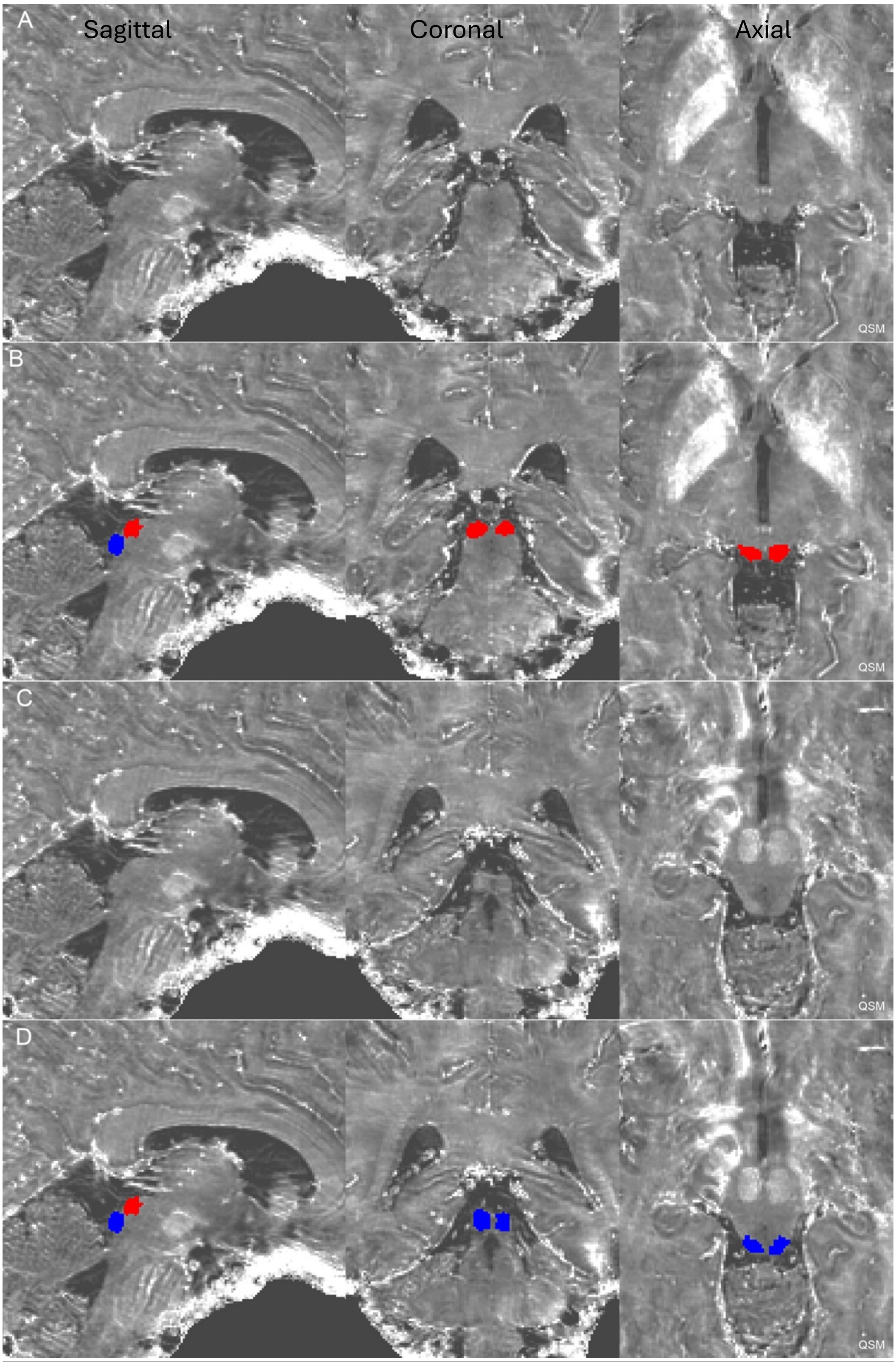

Fig 1. QSM contrasts showing the location of the superior (red) and inferior colliculus (blue) at the dorsal aspect of the brainstem. Note the well-defined borders with the ventricular system facilitating the delineations.

Anterior and posterior commissure (*ac* and *pc*)

The *ac* connects the left and right hemisphere of the brain anterior of the fornical columns. Delineations are performed on the R1 maps. The *ac* can be readily identified in the axial or coronal plane, crossing the midline of the brain through the third ventricle (3V). In the sagittal plane the *ac* has an oval appearance. Laterally, the *ac* runs inferior to the striatum and into the temporal lobe. Our delineations are limited to the part of the anterior commissure in the subcortical region of the brain, and do not include the extension to the temporal lobe. A landmark based cut off is made where the *ac* enters the temporal lobe. The posterior commissure (*pc*, in red) also connects the left and right hemispheres. It is located rostral to the superior colliculi and is located dorsal to the opening of the aqueduct of Sylvius into the 3V. The *pc* is thinner than the *ac*.

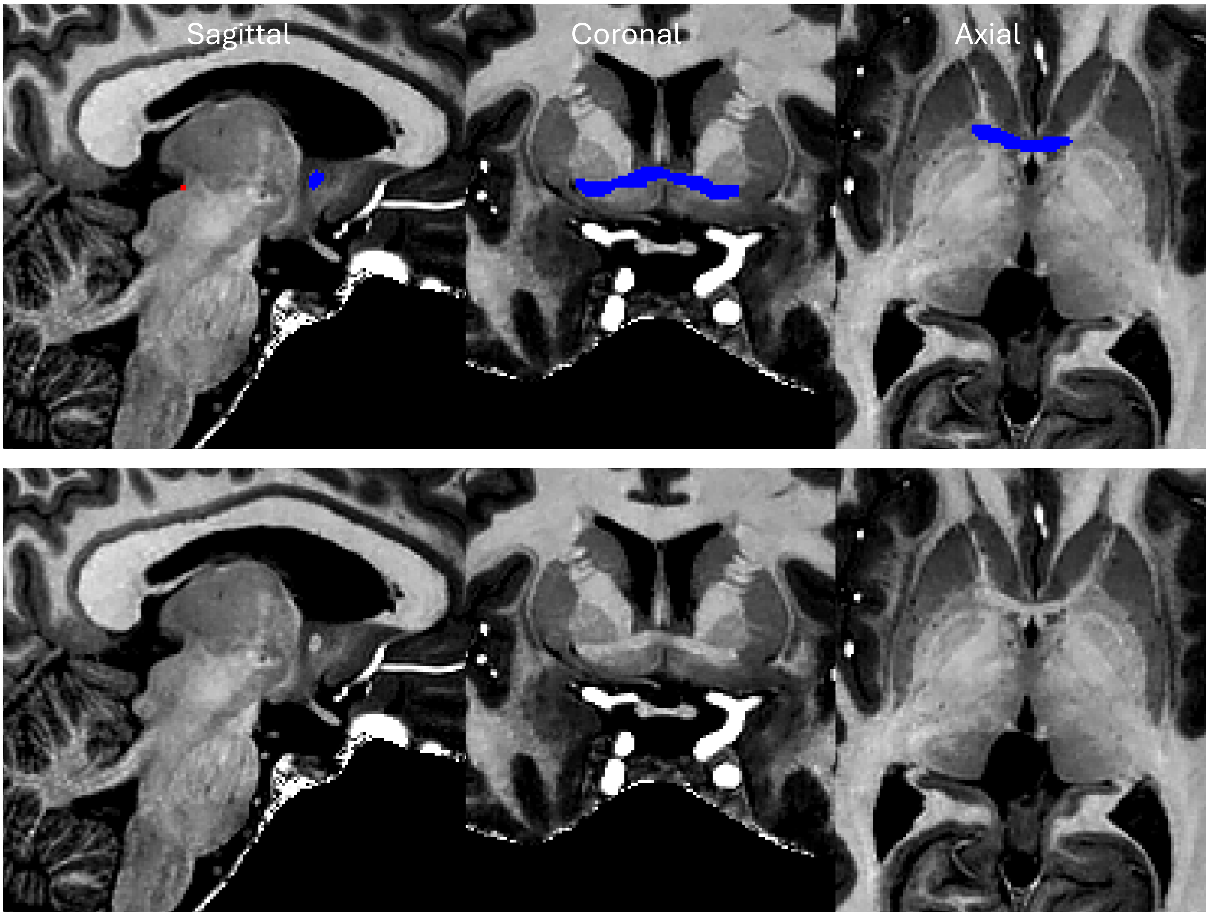

Fig 2: View of the brain showing the starting point for the parcellation. Top row shows the delineated Anterior Commissure (*ac*, blue), bottom row the R1-contrast. In the sagittal view, the Posterior Commissure (*ac*) is visible (red).

We first moved in anterior direction. In multiple cases the *ac* appears to split into two individual structures in rostral views, this is due to the curvature of the structure.

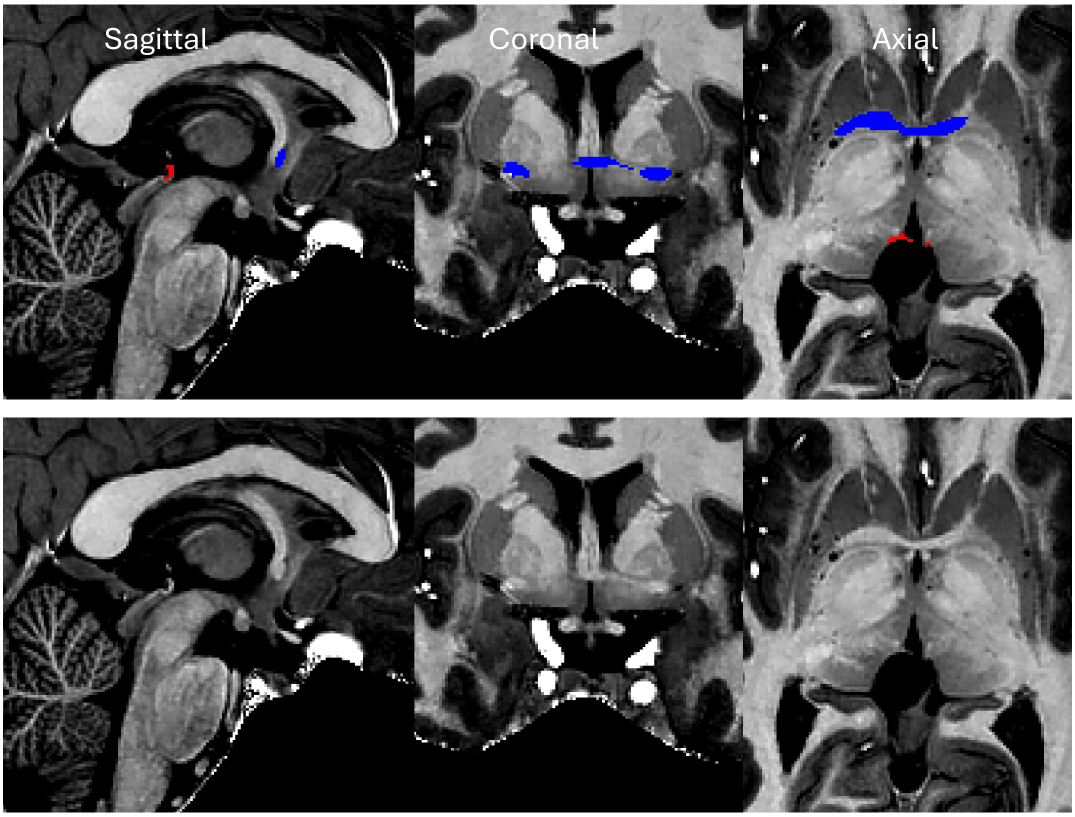

Fig 3: Illustration of the more rostral view of the Anterior Commissure (AC, blue). Note that in the coronal view (middle), the AC does not appear as a continuous structure. In the sagittal view (right), which is explained by the curvature. The Posterior Commissure (PC) is visible in red.

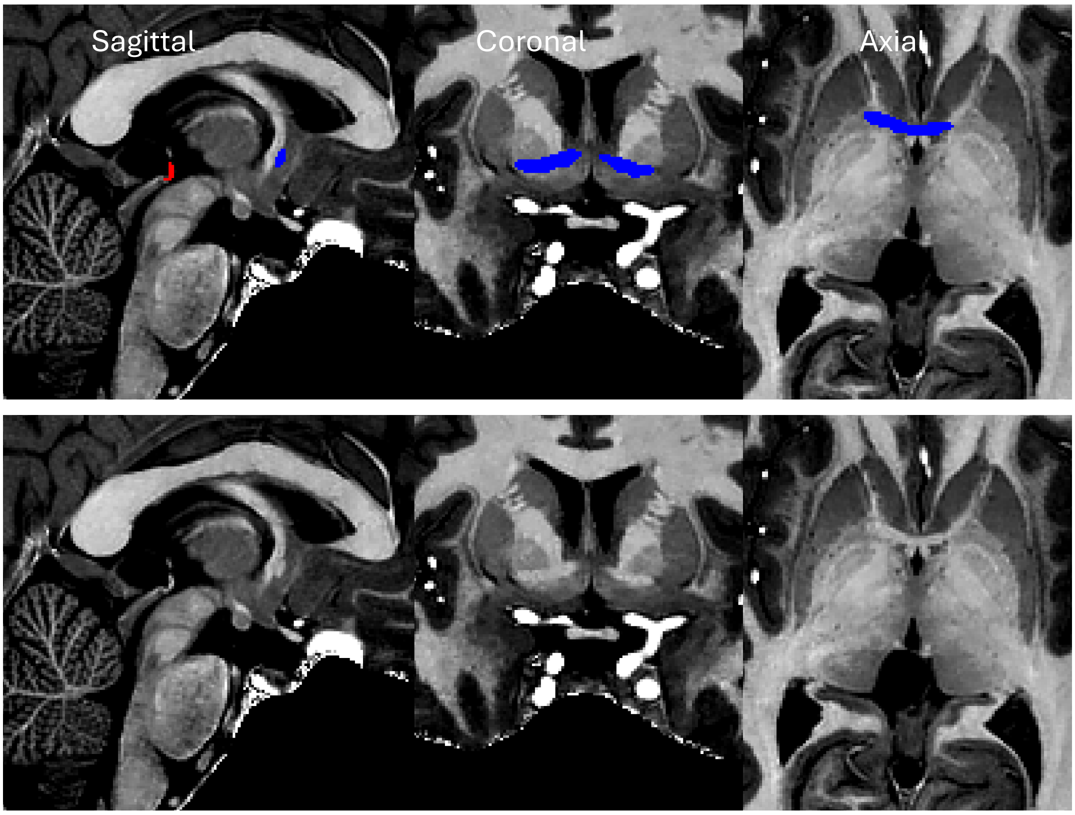

Fig 4: More rostral parts of the Anterior Commissure (AC, blue) show that the left and right side appear detached in the coronal plane. The Posterior Commissure (PC) is visible in red.

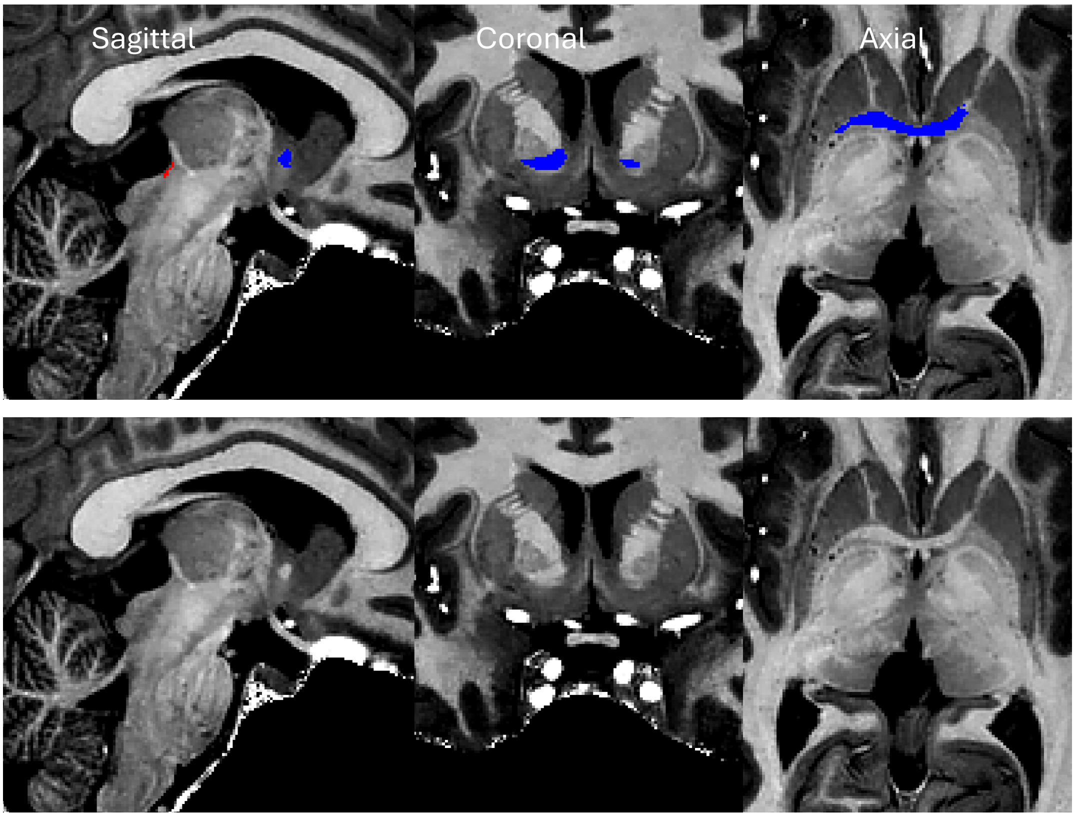

Fig 5: *ac* is slowly disappearing from the rostral views when moving further anterior.

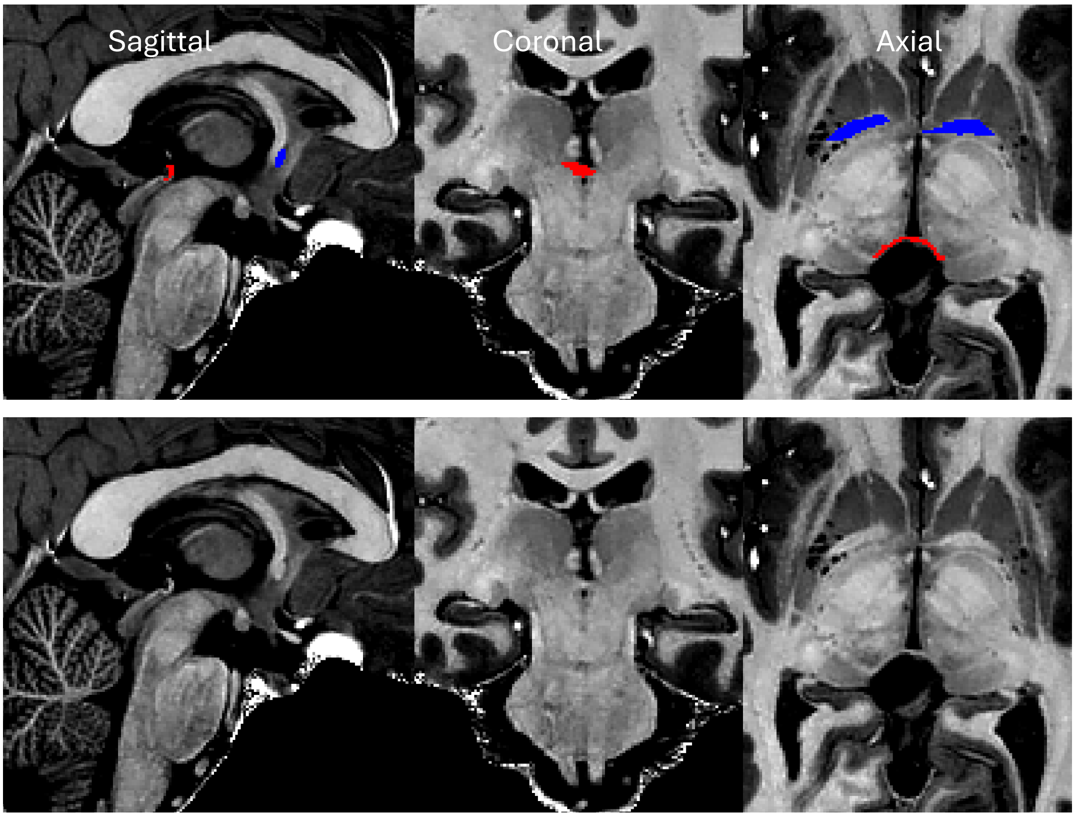

Fig 6: The Posterior Commissure (*pc*) can be identified in the sagittal view (red), and is substantially thinner than Anterior Commissure (*ac*, blue).

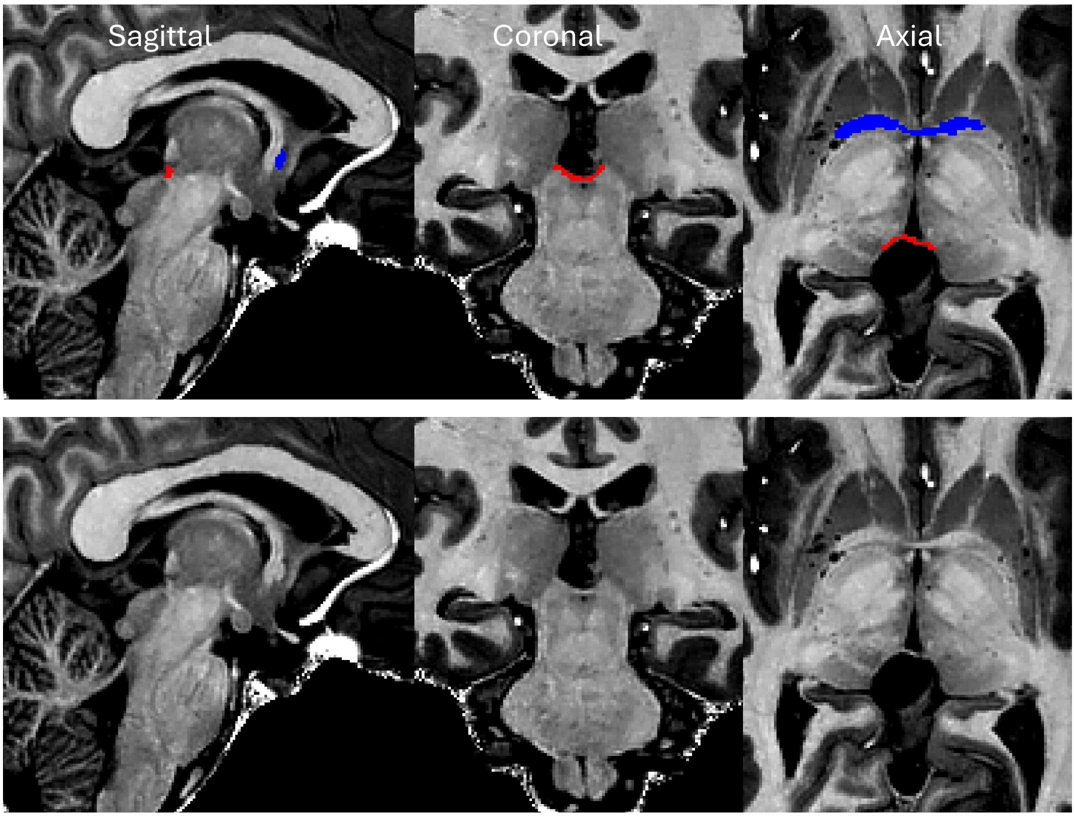

Fig 7: The Posterior Commissure (*pc*, red) can be delineated and the lateral border of the masks are determined by the location of the thalamus (grey matter). The Anterior Commissure (*ac*) is visible in blue.

Cholinergic forebrain nuclei

The cholinergic forebrain nuclei (ChN) show a hyperintense signal on QSM images. We found that the medial septal nucleus and the nucleus of the Diagonal Band of Broca (DBB) were not readily visible on the QSM contrasts. As a result, our delineations contain ChN3-4, but not ChN1-2. Delineations are created on the qMRI 0.05mm slab data, using the R1 and QSM contrasts. The protocol was based on the work by (Zaborszky et al., 2008).

For anatomical orientation, we first locate the anterior commissure in the R1 map, identifying the most anterior part of the commissure. On the QSM, the AC shows a hypointense signal. Inferior to the *ac* a hyperintense signal is present on the QSM, which corresponds to the location of ChN. This area is delineated in the coronal plane guided by the hyperintense contrast, and this outline provides guidance for further delineations in the axial plane. The masking is continued first in the inferior and subsequently in the superior direction, guided by the available contrast. Note that dependent on the orientation of the scan, it may appear as two separate structures in some of the more inferior slices. After completion of the delineations, shape consistency is confirmed in the coronal view.

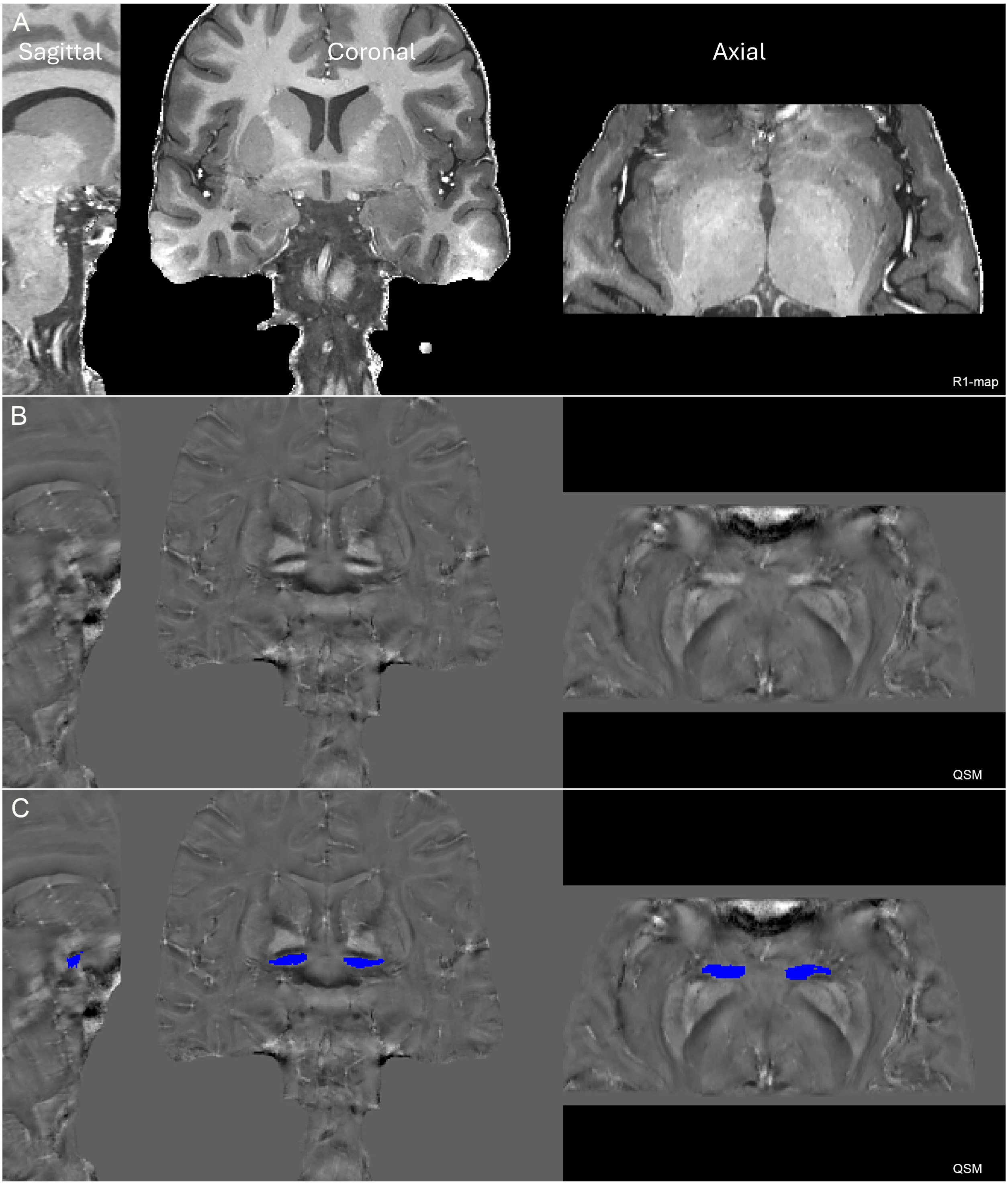

Fig 8: A Showing R1-maps of the 0.05mm isotropic slabs for anatomical orientation and the visualization of the anterior commissure. B Note the hyperintense QSM signal inferior to the commissure corresponding to the cholinergic forebrain nuclei. C Corresponding outline of the Ch4 area.

Lateral habenula

Delineation of the habenular complex in the AHEAD database has been described previously, and was performed on the R1 contrasts of the slab data (Alkemade et al., 2022). In short, the R1 contrast is used to locate the center of the Habenular complex. Delineations are created in the coronal plane, first moving in the anterior and then in the posterior direction. The rostral border is defined at the level where the stria medullaris (sm) is first observed to bulge into the third ventricle. Since sm and the Habenula both show hypointense R1 contrast, it is likely that parts of the sm are included in the delineations. At its central level, the habenula may appear more triangular in some participants, and more elongated in others. The transverse view is used for the separation of the habenula from the fasciculus retroflexus (fr).

Dorsal and medial Raphé nucleus

The Raphé complex consists of a group of nuclei located along the midline of the brainstem (medulla oblongata, pons, and midbrain). These nuclei are small and rich in iron. Using enhanced contrast settings, they can be discerned on the QSM. The reticular complex is located in close lateral proximity, and Raphé nuclei do not extend beyond a few millimeter in lateral direction from the midline. R1 and QSM contrasts are used for anatomical orientation. First the location of the Red Nucleus (RN) is determined. At the center levels of the Red Nucleus in the coronal view, at the midline of the brain the DRN can be discerned as a somewhat hyperintense signal on the QSM contrast. Note that the signal is not as hyperintense as that of the RN, and therefore the QSM contrast requires increasing. In the sagittal view, the MRN is consistently visible using the same contrast settings. The RPo and RMG, Raphé pallidus, (RPa) and Raphé obscurus (Rob) proved to be difficult to delineate in all participants of the training sets. We therefore did not pursue atlasing of these four structures.

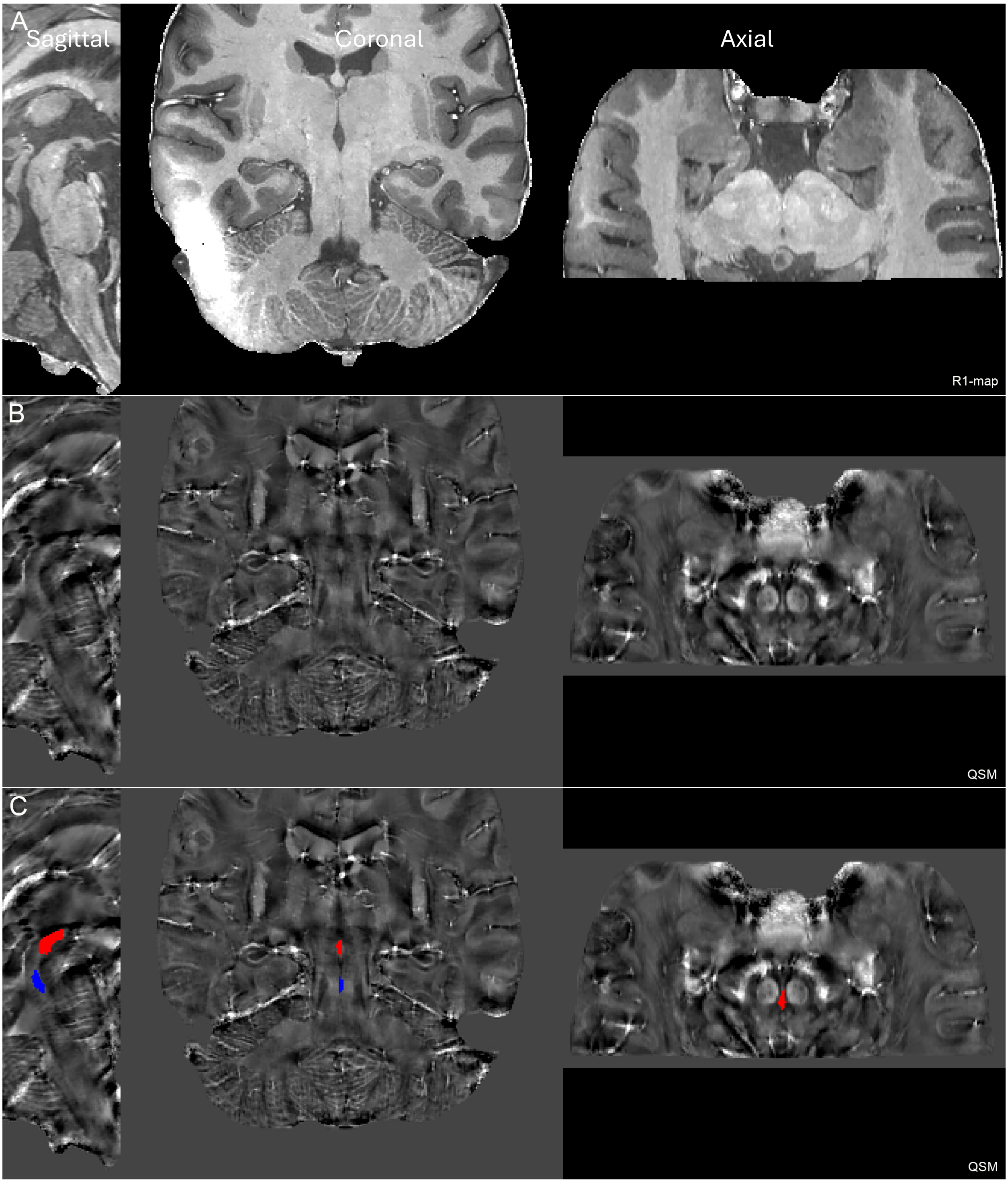

Fig 9: A: R1 map for anatomical orientation. B. QSM contrast showing the location of the RN, and at the midline the starting point for the delineation of the DRN (in red). After delineation of the DRN, the MRN (blue) is outlined.

Hippocampal subfields

An excellent guide for the delineation of the hippocampus has been published by Dalton et al., 2017). Delineations were performed in the coronal plane, and were largely based on anatomical landmarks. The main contrast used was the R1-map. First the DG/CA4 mask was created, followed by the mask that included CA2 and 3. CA2 and 3 were grouped since it was not possible to discern the border between these regions on MRI. Subsequently we delineated CA1. It was challenging to define the anterior border of CA1. After delineation of the uncus, the subiculum was identified in the ventral portion of the hippocampus. It is located medial to CA1. The subiculum and prosubiculum were combined in a single mask, as were the pre and parasubiculum. Finally the uncus was delineated.

Nucleus Accumbens, Caudate and Putamen

We derived masks for the NACC, CAU and PUT from our previous manual delineations (Alkemade et al., 2022). Existing masks covering the whole striatum were used as a basis. The Internal Capsule was identified as an anatomical landmark. A line perpendicular to the base of the *ic* was used as an arbitrary border between the NACC and the CAU and PUT. The CAU and PUT were further separated by the internal capsule, and the striatal bridges between the CAU and PUT were included in neither of the masks.

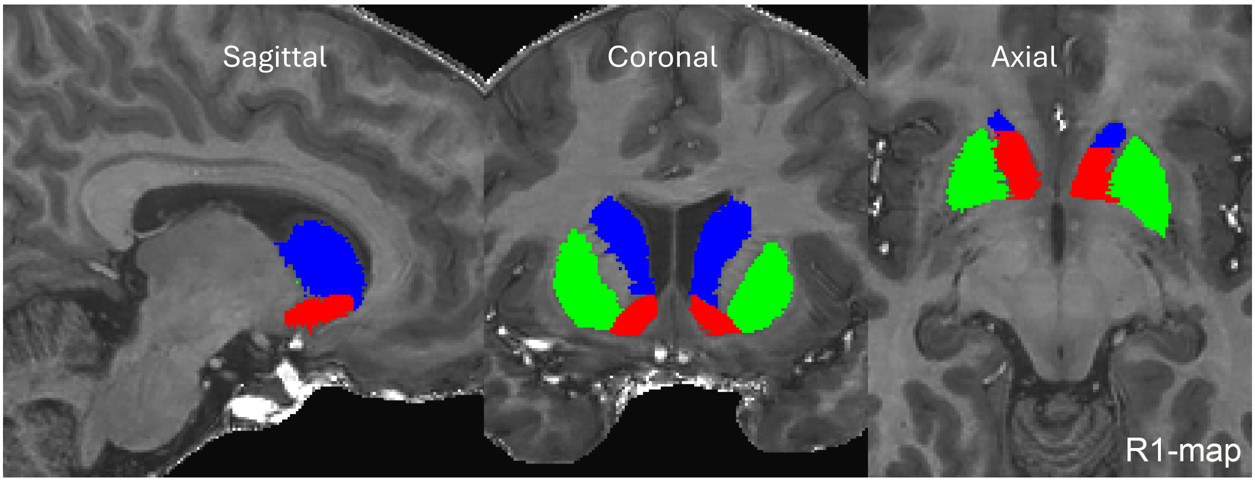

Fig 10: Adapted manual delineation of the striatum, creating separate masks for the Nucleus Accumbens (red), the Caudate (blue) and the Putamen (green).

Alkemade, A., Mulder, M. J., Trutti, A. C., & Forstmann, B. U. (2022). Manual delineation approaches for direct imaging of the subcortex. *Brain Structure and Function*, *227*(1), 219–297. https://doi.org/10.1007/S00429-021-02400-X/FIGURES/35

Dalton, M. A., Zeidman, P., Barry, D. N., Williams, E., & Maguire, E. A. (2017). Segmenting subregions of the human hippocampus on structural magnetic resonance image scans: An illustrated tutorial. *Brain and Neuroscience Advances*, *1*, 239821281770144. https://doi.org/10.1177/2398212817701448

García-Gomar, M. G., Strong, C., Toschi, N., Singh, K., Rosen, B. R., Wald, L. L., & Bianciardi, M. (2019). In vivo Probabilistic Structural Atlas of the Inferior and Superior Colliculi, Medial and Lateral Geniculate Nuclei and Superior Olivary Complex in Humans Based on 7 Tesla MRI. *Frontiers in Neuroscience*, *13*, 764. https://doi.org/10.3389/fnins.2019.00764

Zaborszky, L., Hoemke, L., Mohlberg, H., Schleicher, A., Amunts, K., & Zilles, K. (2008). Stereotaxic probabilistic maps of the magnocellular cell groups in human basal forebrain. *NeuroImage*, *42*(3), 1127–1141. https://doi.org/10.1016/J.NEUROIMAGE.2008.05.055
