# Supplementary material for: Automated parcellation and atlasing of the human subcortex with ultra-high resolution quantitative MRI
Source: Imaging Neurosci (Camb). 2025 Apr 29;3:imag_a_00560. doi: 10.1162/imag_a_00560 (PMC12319765; doi:10.1162/imag_a_00560)
Supplement: Supplement 2 [file imag_a_00560-supp2.pdf]

Supplement 2: Comparison of MASSP and MASSP2.0

Automated parcellation and atlasing of the human subcortex with ultra high-resolution quantitative MRI

Pierre-Louis Bazin[1], Josephine M Groot[2], Steven Miletic[3,4], Lysanne Groenewegen[3], Anne C Trutti[3], Martijn J Mulder[5], Birte U Forstmann[3], Anneke Alkemade[3]

[1]Full brain picture Analytics, Leiden, The Netherlands
[2]Integrative Model-Based Neuroscience Research Unit, University of Amsterdam, Amsterdam, The Netherlands
[3]Cognitive Psychology Unit, Institute of Psychology, Leiden University, The Netherlands
[4] University of Utrecht, Psychology and Social Sciences, Utrecht, The Netherlands
Corresponding author: Anneke Alkemade, Integrative Model-based Cognitive Neuroscience Research Unit, University of Amsterdam, , Nieuwe Achtergracht 129B | Room G0.01, PO box 15926,1001 NK Amsterdam, The Netherlands

j.m.alkemade@uva.nl

Keywords:

Subcortex, 7 Tesla MRI, probabilistic atlasing, automated brain parcellation

Supplementary table 1: Performance comparison between MASSP and MASSP2.0

**Structure**	**Dice overlap**		**dilated Dice overlap**		**boundary distance (mm)**	
	MASSP	*MASSP2.0*	*MASSP*	*MASSP2.0*	*MASSP*	*MASSP2.0*
STN	0.65	0.72	0.92	0.92	0.64	0.54
SN	0.81	0.82	0.98	0.97	0.47	0.46
RN	0.87	0.88	0.99	0.99	0.34	0.33
GPi	0.70	0.77	0.91	0.93	0.80	0.59
GPe	0.80	0.82	0.96	0.95	0.60	0.56
Tha	0.87	0.87	0.97	0.96	0.74	0.75
LV	0.85	0.92	0.93	0.98	1.88	0.58
3V	0.74	0.83	0.89	0.96	0.70	0.47
4V	0.87	0.86	0.95	0.97	0.57	0.53
Amg	0.72	0.76	0.89	0.88	1.03	0.98
ic	0.75	0.78	0.92	0.93	0.89	0.77
VTA	0.57	0.64	0.86	0.89	0.75	0.51
fx	0.53	0.73	0.76	0.94	2.07	0.45
PAG	0.64	0.73	0.90	0.92	0.67	0.51
PPN	0.50	0.59	0.80	0.84	1.04	0.70
Cl	0.49	0.55	0.79	0.83	1.18	0.69

Dice overlap is calculated between the MASSP algorithms and manual delineations. Note that MASSP delineations were based on 0.64 x 0.64 x 0.70 mm^3 scans only, and the MASSP2.0 delineations were performed on the combined information of the 0.64 x 0.64 x 0.70 mm^3 and the 0.5 x 0.5 x 0.5 mm^3.

Supplementary table 2: Performance of MASSP2.0 across the adult lifespan

**Structure**	**Dice overlap (sem)**	**dilated Dice overlap (sem)**	**n**
STN_l	0.58 (0.05)	0.83 (0.04)	11
STN_r	0.64 (0.05)	0.86 (0.04)	11
SN_l	0.81 (0.02)	0.95 (0.01)	11
SN_r	0.81 (0.02)	0.95 (0.02)	11
RN_l	0.85 (0.02)	0.97 (0.01)	11
RN_r	0.87 (0.01)	0.98 (0.01)	11
GPi_l	0.70 (0.02)	0.87 (0.02)	11
GPi_r	0.71 (0.04)	0.87 (0.04)	11
GPe_l	0.78 (0.02)	0.92 (0.01)	11
GPe_r	0.78 (0.01)	0.92 (0.01)	11
Tha_l	0.88 (0.11)	0.96 (0.00)	9
Tha_r	0.88 (0.11)	0.97 (0.00)	9
Amg_l	0.70 (0.04)	0.82 (0.04)	11
Amg_r	0.71 (0.03)	0.83 (0.03)	11

Dice overlap is calculated between the MASSP2.0 algorithm and manual delineations for a subset of structures and participants with an age distribution across the entire adult lifespan.
